# New perspectives for simultaneous attraction of *Chrysoperla* and *Chrysopa* lacewing species for enhanced biological control (Neuroptera: Chrysopidae)

**DOI:** 10.1038/s41598-019-46621-x

**Published:** 2019-07-16

**Authors:** Sándor Koczor, Ferenc Szentkirályi, Miklós Tóth

**Affiliations:** Plant Protection Institute, Centre for Agricultural Research, Hungarian Academy of Sciences, H-1022 Budapest, Herman Ottó út 15. Hungary

**Keywords:** Chemical ecology, Animal behaviour

## Abstract

Green lacewings (Chrysopidae) are important predators of many soft-bodied pest insects, for instance aphids. Previous studies reported attraction of *Chrysoperla carnea* species-complex to a ternary floral bait. The larvae of these lacewings are important generalist predators in agroecosystems, however adults are non-predatory, they feed on pollen, nectar or honeydew. Squalene, a plant originated compound was previously reported to be attractive to the nearctic *Chrysopa nigricornis*. In the current study squalene was tested alone and in combination with the ternary bait in field experiments in Hungary. In our experiments, traps baited with squalene attracted predatory males of *Chrysopa formosa*. Traps baited with squalene and the ternary floral bait attracted adults of both *C*. *formosa* and *C*. *carnea* complex lacewings. To our knowledge this is the first report of a bait combination attractive to both *Chrysoperla* and *Chrysopa* species. This finding is of special interest considering the remarkably different feeding habits of the adults of these lacewings. Potential perspectives in biological control are discussed.

## Introduction

Green lacewings are important predators of many soft-bodied insect pests, especially aphids and other Sternorrhyncha^1^. Among green lacewings, *Chrysoperla* spp. are of special importance in agroecosystems^2,3^. Previous studies reported attraction of *Chrysoperla* spp. to plant originated compounds (e.g.^4,5^). It was also found that a ternary blend, consisting of phenylacetaldehyde, methyl salicylate and acetic acid, was more attractive to *Chrysoperla carnea* species-complex (i.e. *C*. *carnea* complex) than previously published attractants^6^. Apart from attraction, the ternary blend showed strong behavioural activity, females laid eggs in the vicinity of the baits^7–9^. This is of special importance, since larvae of these lacewings are voracious, generalist predators^3^. Nevertheless, adults of these lacewings are not predatory, but feed on pollen, nectar and honeydew, thus are termed palyno-glycophagous^1^.

On the other hand, both adults and larvae of *Chrysopa* spp. are predatory^1^, thus, simultaneous attraction of *Chrysoperla* and *Chrysopa* species could be of potential significance in biological control. However, to our knowledge no such stimulus combination is known. From this viewpoint attractants for *Chrysopa* species and their interactions with *Chrysoperla* attractants are of special interest.

Aphid sex pheromone components were found to attract males of some *Chrysopa* species (e.g.^10–13^). Nevertheless, combination of the ternary floral bait and aphid sex pheromone components resulted in markedly decreased attraction of *Chrysoperla* spp. lacewings to the otherwise highly attractive ternary floral bait^12,14^.

Jones *et al*.^15,16^ found attraction of a nearctic *Chrysopa* species, *C*. *nigricornis* Burmeister, 1839 to traps baited with squalene, a plant originated compound.

The aim of the current study was to test potential attraction of green lacewing species to squalene in Central Europe, with respect to potential interactions with the ternary floral bait.

## Materials and Methods

### Preparation of baits

All synthetic compounds (>90% chemical purity as per the manufacturer) were obtained from Sigma-Aldrich Kft (Budapest, Hungary). Two different formulations were used: the polyethylene bag (PE bag) and the polyethylene vial (PE vial) formulations, both of which have been found to be effective in previous experiments on green lacewings (e.g.^6,12^).

For the PE bag formulation, compounds were loaded onto a 1 cm piece of dental roll (Celluron®, Paul Hartmann AG, Heidenheim, Germany), which was put into a polyethylene bag (ca 1.0 × 1.5 cm) made of 0.02 mm linear polyethylene foil (FS471-072, Phoenixplast BT, Pécs, Hungary). The dispensers were heat sealed.

For the PE vial formulation compounds were loaded into 0.7 ml polyethylene vials with lid (No. 730, Kartell Co., Italy), the lids of the dispensers were closed.

The ternary floral baits were formulated into PE bag dispensers. The baits comprised of three components, i.e. phenylacetaldehyde, acetic acid and methyl salicylate, in a 1:1:1 ratio^6^. The loading of each compound was kept at 100 mg.

For Exp. 1, 100 mg of squalene was formulated into PE bag dispensers. For Exp. 2, loads of 1, 10 or 100 mg and for Exp. 3., 100 mg of squalene were formulated into PE bag dispensers, respectively. For Exp. 4, 100 mg of squalene was formulated into either PE bag or PE vial dispensers.

Both PE bag and PE vial dispensers were attached to 8 × 1 cm plastic handles for easy handling when assembling the traps. For storage, baits were wrapped singly in pieces of aluminium foil and stored at −18 °C until used. In the field, baits were changed at 3 to 4 week intervals, as previous experience showed that they do not lose their attractiveness during this period.

### Field tests

Field experiments were performed from 2013 to 2015 in a mixed orchard in Hungary at Halásztelek (47°21′11″ N, 19°0′20″E).

For the experiments CSALOMON^®^ VARL + funnel traps were used (produced by Plant Protection Institute, CAR, HAS, Budapest, Hungary), which proved to be suitable for catching green lacewings^6,12,14^.

In all field experiments one replicate of each treatment was incorporated into a block, so that individual treatments were 5–8 m apart. Within each block the arrangement of treatments was randomized. Distance between blocks was 15–20 meters. Traps were suspended in the canopy of trees at a height of ca 1.5–1.8 m. As a rule, traps were checked twice weekly.

Description of experiments:

Experiment 1: the objective was to test the potential attraction of green lacewings to squalene. The following treatments were included: ternary floral bait alone, squalene bait alone, and unbaited traps. The experiment was run with 6 blocks from June 5 to July 29, 2013.

Experiment 2: the objective was to test attraction of *C*. *formosa* to different doses of squalene. The following treatments were included: traps baited with either 1, 10 or 100 mg of squalene, and unbaited traps. The experiment was run with 7 blocks from May 29 to August 7, 2014.

Experiment 3: the objective was to test for potential interactions between the ternary floral and squalene baits. The following treatments were included: ternary floral bait alone, squalene bait alone, both ternary floral and squalene baits, and unbaited traps. The experiment was run with 7 blocks from May 29 to August 7, 2014.

Experiment 4: in this experiment different formulations of squalene were tested, the following treatments were included: squalene in PE bag formulation, squalene in PE vial formulation, and unbaited traps. The experiment was run with 5 blocks from June 4 to July 27, 2015.

The green lacewings caught were taken to the laboratory and determined to species level according to relevant taxonomic works on Chrysopidae^17–20^.

### Statistics

For all experiments, catches per trap were summed and data were tested for normality by Shapiro-Wilk test. Since none of the experimental data was found to be normally distributed, catches were analyzed by Kruskal-Wallis test. Level of significance was set at p = 0.05. If significant differences were found, differences between treatments were evaluated by pairwise Wilcoxon rank sum test with Bonferroni correction. Dose-response correlations were tested by Spearman’s rank correlation test. All statistical procedures were conducted using the software R^21^.

## Results

In the experiments *Chrysopa formosa* Brauer, 1850 and *Chrysoperla* spp. were caught in sufficient numbers for further analysis. Among *Chrysoperla* spp., *Chrysoperla carnea* s. str. (Stephens, 1836), *Chrysoperla lucasina* (Lacroix, 1912) and *Chrysoperla pallida* Henry, Brooks, Duelli & Johnson, 2002 were caught, however, since no differences were observed between these species in the analyses, these were treated as *C*. *carnea* complex.

**Figure 1:**
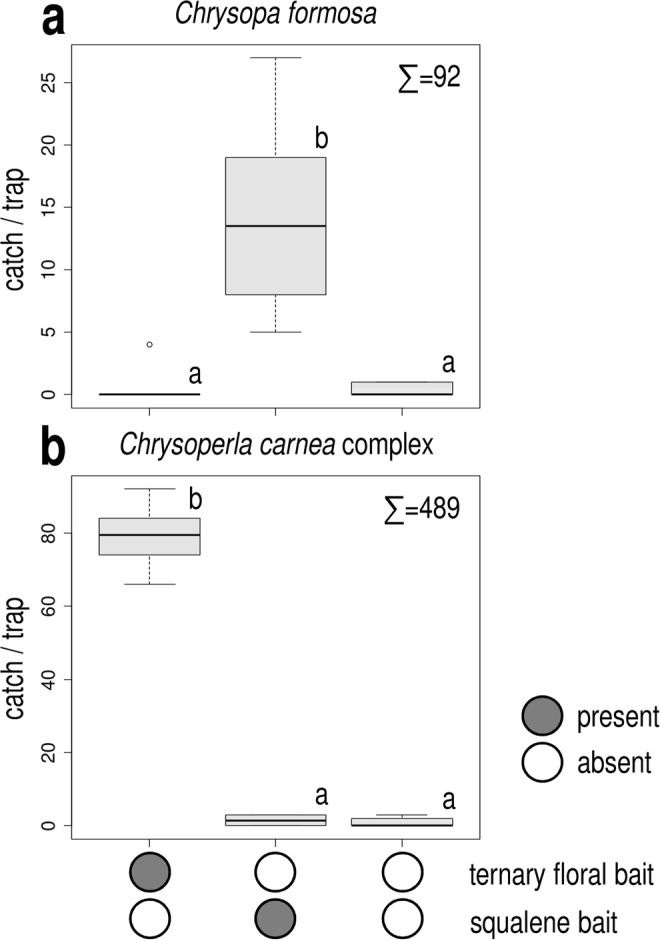
Catches of *Chrysopa formosa* (**a**) and *Chrysoperla carnea* species-complex (**b**) in funnel traps baited with squalene or the ternary floral bait and in unbaited traps (Experiment 1). Catches marked with the same letter are not significantly different within one diagram (Kruskal-Wallis test, followed by pairwise comparisons by Wilcoxon rank sum test with Bonferroni correction at p = 0.05) Σ = total caught in test.

In Exp. 1, squalene attracted significantly more *C*. *formosa* than other treatments (Fig. 1a), which caught very low numbers and did not differ significantly among themselves. Captured individuals consisted almost exclusively of males, only one female was caught. As for the *C*. *carnea* complex, only the ternary floral bait attracted more individuals than unbaited traps (Fig. 1b). Both males and females were attracted.

In Exp. 2, increasing doses of squalene attracted increasing numbers of *C*. *formosa* (Table 1), the correlation between dose and catches was positive and significant (Spearman’s rho = 0.85, p < 0.001). Traps baited with 100 mg of squalene attracted more *C*. *formosa*, than other treatments (Table 1). Only males were caught.

**Table 1:** Catches of *Chrysopa formosa* and *Chrysoperla carnea* species-complex in funnel traps baited with different doses of squalene and in unbaited traps (Experiment 2).

bait load	catch/trap (mean ± SE)			
	*Chrysopa formosa*		*Chrysoperla carnea* complex	
	males	females	males	females
1 mg squalene	0 ± 0a	0 ± 0a	0.4 ± 0.3a	1.1 ± 0.3b
10 mg squalene	0.7 ± 0.4a	0 ± 0a	0.9 ± 0.3a	0.3 ± 0.2ab
100 mg squalene	5.4 ± 2.5b	0 ± 0a	0.9 ± 0.4a	0 ± 0a
no bait	0 ± 0a	0 ± 0a	0.1 ± 0.1a	0.3 ± 0.2ab
total caught	43	0	16	12

Catches marked with the same letter are not significantly different (Kruskal-Wallis test, followed by pairwise comparisons by Wilcoxon rank sum test with Bonferroni correction at p = 0.05).

As for the *C*. *carnea* complex very few individuals were caught, treatments generally did not differ significantly (Table 1) with the exception of a slight significance of females caught by 1 mg vs. 100 mg (p = 0.019, Wilcoxon rank sum test with Bonferroni correction).

**Figure 2:**
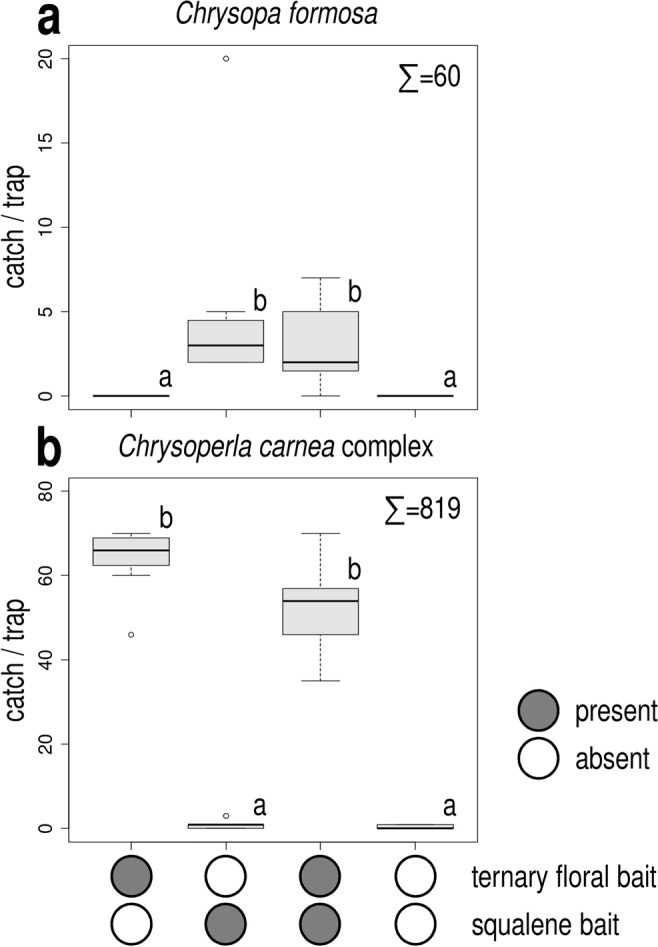
Catches of *Chrysopa formosa* (**a**) and *Chrysoperla carnea* species-complex (**b**) in funnel traps baited with squalene, with ternary floral bait, both and in unbaited traps (Experiment 3). Catches marked with the same letter are not significantly different within one diagram (Kruskal-Wallis test, followed by pairwise comparisons by Wilcoxon rank sum test with Bonferroni correction at p = 0.05) Σ = total caught in test.

In Exp. 3, only treatments containing squalene attracted more *C*. *formosa* than unbaited traps, catches of these treatments did not differ significantly, irrespective of the presence or absence of the ternary floral bait (Fig. 2a). Only male *C*. *formosa* were caught.

As for *C*. *carnea* complex, only treatments containing the ternary floral bait attracted more individuals than unbaited traps, irrespective of the presence of squalene baits (Fig. 2b). Both males and females were attracted.

In Exp. 4, relatively few *C*. *formosa* were caught, however squalene formulated into PE vial dispensers attracted significantly more males, than other treatments, which did not differ significantly (Table 2). Only one female *C*. *formosa* was caught. Only few *C*. *carnea* complex lacewings were caught in the traps, and their catches did not differ significantly among treatments.

**Table 2:** Catches of *Chrysopa formosa* and *Chrysoperla carnea* species-complex in funnel traps baited with different formulations of squalene and in unbaited traps (Experiment 4).

trap bait	catch/trap (mean ± SE)			
	*Chrysopa formosa*		*Chrysoperla carnea* complex	
	males	females	males	females
squalene in PE vial	6.4 ± 1.7b	0.0 ± 0.0a	0.2 ± 0.2a	0.2 ± 0.2a
squalene in PE bag	0.8 ± 0.2a	0.2 ± 0.2a	0.0 ± 0.0a	0.4 ± 0.2a
no bait	0.0 ± 0.0a	0.0 ± 0.0a	0.2 ± 0.2a	0.6 ± 0.2a
total caught	36	1	2	6

Catches marked with the same letter are not significantly different (Kruskal-Wallis test, followed by pairwise comparisons by Wilcoxon rank sum test with Bonferroni correction at p = 0.05).

## Discussion

The current results have shown that squalene is attractive to males of *C*. *formosa*. To our knowledge this is the first report on the attraction of a Palaearctic green lacewing species to this compound.

Squalene was found to be produced in elevated quantities in response to leafminer damage in apple leaves^22^. Furthermore, it elicited oviposition response from female parasitoids (Hymenoptera: Braconidae), however, it was not attractive in olfactometer assays^22^. Furthermore, squalene has also been reported as a semiochemical for ticks^23^.

In our experiments the vast majority of the attracted *C*. *formosa* were males, female catches were negligible. This is in accordance with the findings of Jones *et al*.^15,16^ on the nearctic *C*. *nigricornis*. We cannot offer an explanation off hand on this highly interesting issue, since squalene is a plant orinigated compound emitted upon pest damage^22^ so sex-specific attraction of males is surprising. Interestingly aphid sex pheromones were also found to attract almost exclusively males of *Chrysopa* spp. (e.g.^11^). Aldrich *et al*.^24^ found that aphid sex pheromone components could act as precursors in pheromone synthesis of green lacewings, however, these compounds are synthesized by sexual forms of aphids^25^ which are present late in the season, when green lacewings are in general inactive mostly as preimaginal forms preparing to overwinter^26^. Further studies may shed more light on the ecological background of the sex-specific attraction to these semiochemicals.

Attraction of male *Chrysopa* have previously been reported, for instance to aphid sex pheromone components (e.g.^11–14^) and to (1*R*,2*S*,5*R*,8*R*)-iridodial, a compound identified from males of two nearctic *Chrysopa* species^13^. Nevertheless, in view of biological control, semiochemicals attracting *Chrysopa* females would be highly advantageous. Chauhan *et al*.^27^ reported higher abundance of *Chrysopa oculata* Say, 1839 males and females in a 5 meter radius area around iridodial baits, as compared to unbaited control. At the same time, the authors noted that in another experiment iridodial-baited traps caught almost exclusively males^27^. These results suggest iridodial to be a potent attractant for *C*. *oculata* males, but certainly a different mode of action (e.g. arrestant) for females^28^. Furthermore, it should be considered that males aggregating around the baits possibly also affected aggregation of females, either by chemical or by vibrational signals, which are also known to be important in communication of green lacewings^29^. Chauhan *et al.*^27^. hypothesised that male *C*. *oculata* form leks, which would be in accordance with higher density of males resulting in increased local abundance of females.

The ternary floral bait attracts both sexes of *C*. *carnea* complex (e.g.^6^), furthermore, females were found to lay their eggs in the vicinity of the baits^7–9^. Since predatory *C*. *carnea* complex larvae have more limited mobility, they will search for prey in the vicinity, which offers potentials for targeted biological control. On the other hand adults of *C*. *carnea* complex are not predatory^1^, thus, from this respect simultaneous attraction of predatory adults of *C*. *formosa* could offer new perspectives for an improved biological control.

In previous field experiments conducted in Hungary, aphid sex pheromone components (1*R*,4a*S*,7*S*,7a*R*)-nepetalactol and (4a*S*,7*S*,7a*R*)-nepetalactone attracted males of *C*. *formosa*, however, in combination with the ternary floral bait attraction of *C*. *carnea* complex lacewings was considerably decreased^12,14^.

Methyl salicylate, a herbivory-induced plant volatile was found to increase catches of *C*. *oculata*, *C*. *nigricornis* and *Chrysopa septempunctata* Wesmael, 1841 (=*Chrysopa pallens* Rambur, 1838 according to^30^) males in combination with iridodial (e.g.^15,31^). Methyl salicylate is also an important component of the ternary floral bait, which attracts *Chrysoperla* spp.^6^. Nevertheless, in the current study, combination of squalene and the ternary floral bait did not result in increased catches of *C*. *formosa* as compared to traps baited with squalene only.

In conclusion, in the course of the current study, traps baited with both the ternary floral bait and squalene together attracted both *C*. *carnea* complex and *C*. *formosa* lacewings at the same time without significant decrease of catches of either taxa as compared to individual stimuli. To our knowledge this is the first lure combination that is attractive to both *Chrysoperla* and *Chrysopa* lacewings. Thus, this stimulus combination is attractive to lacewing adults with remarkably different feeding habits (palyno-glycophagous and predatory). This finding may be of benefit in biological control as well, since simultaneous attraction of these taxa with different life history traits may offer more efficient control of harmful insects through predation by both larvae (*Chrysoperla* spp.) and adults (*C*. *formosa* males) of lacewings attracted.

## References

[1] Canard, M. Natural food and feeding habits of lacewings. In: Lacewings in the crop environment (eds McEwen, P. K., New, T. R. & Whittington, A.), 116–128 (Cambridge University Press, Cambridge, UK, 2001).

[2] Stelzl M, Devetak D (1999). Neuroptera in agricultural ecosystems. Agricult. Ecosys. Environ..

[3] Pappas ML, Broufas GD, Koveos DS (2011). Chrysopid predators and their role in biological control. J. Entomol..

[4] Flint HM, Salter SS, Walters S (1979). Caryophyllene: an attractant for the green lacewing. Environ. Entomol..

[5] Tóth M (2006). Phenylacetaldehyde: A chemical attractant for common green lacewings (*Chrysoperla carnea* s.l., Neuroptera: Chrysopidae). Eur. J. Entomol..

[6] Tóth M (2009). Optimization of a phenylacetaldehyde-based attractant for common green lacewings (*Chrysoperla carnea* s.l., Neuroptera: Chrysopidae). J. Chem. Ecol..

[7] Jaastad G, Hatleli L, Knudsen GK, Tóth M (2010). Volatiles initiate egg laying in common green lacewings. IOBC/wprs Bull..

[8] Koczor S, Knudsen GK, Hatleli L, Szentkirályi F, Tóth M (2015). Manipulation of oviposition and overwintering site choice of common green lacewings with synthetic lure (Neuroptera: Chrysopidae). J. Appl. Ent..

[9] Koczor S, Szentkirályi F, Fekete Z, Tóth M (2017). Smells good, feels good: oviposition of *Chrysoperla carnea*-complex lacewings can be concentrated locally in the field with a combination of appropriate olfactory and tactile stimuli. J. Pest. Sci..

[10] Boo KS, Chung IB, Han KS, Pickett JA, Wadhams LJ (1998). Response of the lacewing *Chrysopa cognata* to pheromones of its aphid prey. J. Chem. Ecol..

[11] Hooper AM (2002). Characterization of (1*R*,4*S*,4a*R*,7*S*,7a*R*)-dihydronepetalactol as a semiochemical for lacewings, including *Chrysopa* spp. and *Peyerimhoffina gracilis*. J. Chem. Ecol..

[12] Koczor S (2010). Attraction of *Chrysoperla carnea* complex and *Chrysopa* spp. lacewings (Neuroptera: Chrysopidae) to aphid sex pheromone components and a synthetic blend of floral compounds in Hungary. Pest. Manag. Sci..

[13] Aldrich J, Zhang QH (2016). Chemical ecology of Neuroptera. Annu. Rev. Entomol..

[14] Koczor S, Szentkirályi F, Pickett JA, Birkett MA, Tóth M (2015). Aphid sex pheromone compounds interfere with attraction of common green lacewings to floral bait. J. Chem. Ecol..

[15] Jones VP (2011). Evaluation of herbivore-induced plant volatiles for monitoring green lacewings in Washington apple orchards. Biol. Control..

[16] Jones VP (2016). Using plant volatile traps to develop phenology models for natural enemies: An example using *Chrysopa nigricornis* (Burmeister) (Neuroptera: Chrysopidae). Biol. Control..

[17] Aspöck, H., Aspöck, U. & Hölzel, H. Die Neuropteren Europas. Eine zusammenfassende darstellung der systematik, ökologie und chorologie der Neuropteroidea (Megaloptera, Raphidioptera, Planipennia) Europas. 2 volumes (Goecke & Evers, Krefeld, 1980).

[18] Henry CS, Brooks SJ, Johnson JB, Duelli P (1996). *Chrysoperla lucasina* (Lacroix): a distinct species of green lacewing, confirmed by acoustical analysis (Neuroptera: Chrysopidae). Syst. Entomol..

[19] Henry CS, Brooks SJ, Duelli P, Johnson JB (2002). Discovering the true *Chrysoperla carnea* (Stephens) (Insecta: Neuroptera: Chrysopidae) using song analysis, morphology, and ecology. Ann. Entomol. Soc. Amer..

[20] Thierry D, Cloupeau R, Jarry M, Canard M (1998). Discrimination of the West-Palearctic *Chrysoperla* Steinmann species of the *carnea* Stephens group by means of claw morphology (Neuroptera, Chrysopidae). Acta Zool. Fennica.

[21] R Core Team, R. A language and environment for statistical computing. R Foundation for Statistical Computing (2016).

[22] Dutton A, Mattiacci L, Amadò R, Dorn S (2002). A novel function of the triterpene squalene in a tritrophic system. J. Chem. Ecol..

[23] Yoder JA, Pollack RJ, Spielman A (1993). An ant-diversionary secretion of ticks: First demonstration of an acarine allomone. J. Insect Physiol..

[24] Aldrich JR, Chauhan K, Zhang QH (2016). Pharmacophagy in green lacewings (Neuroptera: Chrysopidae: *Chrysopa* spp.)?. PeerJ.

[25] Hardie J (1990). Aphid sex pheromone components: age-dependent release by females and species-specific male response. Chemoecology.

[26] Canard M (1998). Life history strategies of green lacewings in temperate climates: a review (Neuroptera, Chrysopidae). Acta Zool. Fennica.

[27] Chauhan KR, Levi V, Zhang QH, Aldrich JR (2007). Female goldeneyed lacewings (Neuroptera: Chrysopidae) approach but seldom enter traps baited with the male-produced compound iridodial. J. Econ. Ent..

[28] Dethier VG, Barton Browne L, Smith CN (1960). The designation of chemicals in terms of the responses they elicit from insects. J. Econ. Ent..

[29] Henry, C. S. Acoustic communication in neuropterid insects. In: *Insect sounds and communication: physiology*, *behaviour*, *ecology*, *and evolut*ion. (eds Drosopoulos, S. & Claridge, M. F.) 153–166 (CRC Press, Taylor & Francis Group, Boca Raton, London, New York 2006).

[30] Aspöck, H., Hölzel, H. & Aspöck, U. Familie Chrysopidae. In: *Kommentierter Katalog der Neuropterida* (*Insecta: Raphidioptera*, *Megaloptera*, *Neuroptera*) der Westpaläarktis. (eds Aspöck, H., Hölzel, H. & Aspöck, U.) 69–124 (Denisia 02, Biologiecentrum des Oberösterreichischen Landesmuseums, Linz, Austria 2001).

[31] Zhang QH, Sheng M, Chen G, Aldrich JR, Chauhan KR (2006). Iridodial: a powerful attractant for the green lacewing, *Chrysopa septempunctata* (Neuroptera: Chrysopidae). Naturwissenschaften.

